# Cellulose-Based Hydrogels as Sustained Drug-Delivery Systems

**DOI:** 10.3390/ma13225270

**Published:** 2020-11-21

**Authors:** Diana Elena Ciolacu, Raluca Nicu, Florin Ciolacu

**Affiliations:** 1 “Petru Poni” Institute of Macromolecular Chemistry, 700487 Iasi, Romania; nicu.raluca@icmpp.ro; 2 Natural and Synthetic Polymers Department, “Gheorghe Asachi” Technical University of Iasi, 700050 Iasi, Romania

**Keywords:** hydrogels, cellulose, stimuli-responsive hydrogels, thermo-responsive, pH-responsive, drug delivery systems, target therapy

## Abstract

Hydrogels, three-dimensional (3D) polymer networks, present unique properties, like biocompatibility, biodegradability, tunable mechanical properties, sensitivity to various stimuli, the capacity to encapsulate different therapeutic agents, and the ability of controlled release of the drugs. All these characteristics make hydrogels important candidates for diverse biomedical applications, one of them being drug delivery. The recent achievements of hydrogels as safe transport systems, with desired therapeutic effects and with minimum side effects, brought outstanding improvements in this area. Moreover, results from the utilization of hydrogels as target therapy strategies obtained in clinical trials are very encouraging for future applications. In this regard, the review summarizes the general concepts related to the types of hydrogel delivery systems, their properties, the main release mechanisms, and the administration pathways at different levels (oral, dermal, ocular, nasal, gastrointestinal tract, vaginal, and cancer therapy). After a general presentation, the review is focused on recent advances in the design, preparation and applications of innovative cellulose-based hydrogels in controlled drug delivery.

## 1. Introduction

The pharmaceutical field has experienced progressive development in the treatment of human diseases, the most recent achievement being the administration of biomolecules (drugs, proteins, etc.) by biomaterial carriers. The development of these carriers made the drugs release possible in certain places of the human body inaccessible by classical administration methods, allowing them to reach the target organs safely, without causing any harm [1].

It is crucial to control the release of drugs, as the pharmacological purpose is not achieved in the case of a rapid release. An “ideal” drug carrier system should deliver an exact amount of drug, at a certain preplanned rate, in order to provide the required drug level for treatment [2]. Thus, by using these systems, the bioavailability of the drugs is improved, the drug concentration is maintained relatively constant during the treatment and, last but not least, undesired side effects are considerably reduced by avoiding some issues such as the absorption in the gastrointestinal tract (GIT) and the hepatic first-pass metabolism [3].

In the last three decades, significant advances have been made in the controlled delivery of drugs, with the main focus on (i) the design of cost-effective drug delivery systems and (ii) non-traditional routes with self-adjusting delivery [4]. In this regard, drug delivery has had to face two big challenges: the zero-order release for longer time and also the controlled release as a response to different external stimuli [5].

The classical drug-delivery systems have many limitations, such as (i) an improper time of drug release, (ii) an ineffective delivery of the drug toward the specific location, along with (iii) eventual toxic side effects on the body [6].

In this regard, the new trends are oriented towards the development of controlled drug-delivery systems capable of meeting the following requirements [7]:

- the improvement of the targeted delivery of drugs to the specific site;
- correlation of the drug release process with the patient’s circadian rhythm, a cycle of approximately 24 h that takes place at the biochemical, physiological and behavioral level, in response of the body to the light-dark alternation;
- optimization of the water-soluble drugs release (reducing the release time);
- increasing the bioavailability of medicines with a low solubility rate in water;
- a carefully delivery control of the highly toxic drugs;
- the use of more drugs in the same system;
- improving the elimination of toxic compounds obtained from drug metabolism (fast elimination).

This review aims to highlight the recent applications of cellulose-based hydrogels, including native cellulose and cellulose derivatives, as drug-delivery systems. Recent literature has been cited, in the first part of the review, to summarize the sites used for drug delivery, the properties of hydrogels suitable for encapsulation, and the main release mechanisms of drugs within the three-dimensional (3D) matrix. The last part of the review deals exclusively with the applications of cellulose-based hydrogels in controlled drug delivery, alone or in combination with other synthetic/natural polymers.

## 2. Controlled Drug-Delivery Locations

### 2.1. Dermal and Transdermal Drug Delivery

Dermal and transdermal administration has become an attractive alternative to official routes, such as oral and parenteral, due to the fact that the skin is an easily accessible organ and is also a way of administering drugs that do not involve pain [8].

However, there are differences in the way drugs are administered for these two routes:

- dermal administration—requires the crossing of the drug through the outer layer of the skin (*stratum corneum*), providing its location in the underlying layers of the skin;
- transdermal delivery—the drug is transported to the skin dermis, followed by its access to the systemic circulation.

Transdermal drug delivery presents some benefits, such as an extended duration of drug delivery at a constant rate, a quick stop of drug administration by simply withdrawing the device, is suitable for self-administration and, a very important aspect, the drug can avoid the hepatic first-pass metabolism and gastro-intestinal incompatibility [8,9].

The administration of dermal drugs is used either to disinfect the skin or to treat it, although there are severe cases, such as the treatment of burns, ulcers or wounds, in which the therapy of the disease is difficult to achieve [1]. A possible treatment for open wounds is the use of hydrogels, as it is important to maintain a moist environment during the healing process of the tissue. It is well known that the moist environment hinder tissue dehydration, stimulates the regeneration of epithelialization and granulation tissue and protects the tissue against microorganisms [3]. Another option of using hydrogels is to treat inflammation or various types of skin diseases (eczema, psoriasis, etc.) that require a system that can incorporate a specific drug and release it through percutaneous penetration. The use of hydrogels in transdermal delivery may offer the benefit of a constant rate of drugs delivery, for an extended duration and, in addition, due to its high-water content, swollen hydrogels may provide a better environment for tissue repair compared with conventional ointments and patches [1,3,10].

Recent research on the utilization of hydrogels in the transdermal administration of the drug has focused on processes such as iontophoresis and electroporation, both of which are used to improve the permeability of various products (hormones or nicotine) [1].

### 2.2. Ocular Drug Delivery

The ocular route of administration of drugs is used only for the treatment of local ocular diseases [9]. However, during the delivery process of the eye drugs, the solution in the form of eye drops tends to be quickly removed from the eye due to eye protection mechanisms, which involve tear drainage, blinking and low corneal permeability [1,11]. This will result in a short release time of the drug, and so a limited absorption of it, which implies reduced or even no therapeutic effect [12]. Under these conditions, it was found that approximately 95% of the drug amount is lost by tear drainage, allowing only 5% of the drug to reach the intraocular tissue [13].

The treatment of ocular pathologies could be significantly improved by prolonging the contact time between drugs and cornea. In this regard, various formulations for ophthalmic treatment have been designed to meet the requirements of having a longer contact time with the ocular surface and also, to present a slower release of drugs [11].

Recently, the use of hydrogels in ocular administration has been considered appealing, due to the fact that they are materials resistant to eye drainage and are an attractive alternative to conventional delivery formulations, such as suspensions or ointments, which can create unpleasant sensations or even irritation, due to their semisolid nature. Moreover, the hydrogels that form in situ are even more attractive due to the possibility of dosing them in the initial liquid state, so that when passing into the gel form, they have a prolonged drug retention capacity [1].

### 2.3. Nasal Drug Delivery

Nasal administration is an attractive choice over the less pleasant or even traumatic parenteral route, or to the oral one, that can offer an unwanted low bioavailability [14]. Nasal administration is recommended by several excellent features, such as: (i) large surface area of the nasal cavity, (ii) rich vascularization of the nasal submucosa, (iii) high permeability of the nasal epithelium and high blood flow, both determining a rapid absorption of the drug, and (iv) avoidance of the hepatic first-pass metabolism [9]. Usually, the nasal cavity is the pathway for the local treatment of nasal diseases, such as rhinitis or nasal congestion, but in recent years, it has become a perspective for the delivery of drugs in the systemic treatments [14].

### 2.4. Gastrointestinal Tract Drug Delivery

Oral administration of drugs is one of the most widely used methods of delivery of therapeutic drugs, which is characterized both by an easy means of delivery of various drugs and by low cost for local or systemic treatments [9,15].

The gastrointestinal tract (GIT) is the best known and most complex way to deliver drugs that has the advantage of a convenient mode of drugs administration and of a large area of systemic absorption, but which require special attention and specific studies for each component organ [1]. GIT is the tract that extends from the mouth to the anus and is composed of several organs, such as: mouth, esophagus, stomach, small intestine and large intestine. The small intestine contains three distinct regions, such as the duodenum, jejunum, and ileum, while the large intestine consists of the cecum, colon (ascending colon, transverse colon, and descending colon), rectum, and anal canal. A detailed presentation of the pH values for each component part of the GIT tract, highlighting of the values for children and adults, is presented in Table 1 [16,17].

Orally administered drugs must overcome several obstacles to eventually reach the bloodstream. For this, the drug must withstand the acidic pH of the stomach, to also resist to the passing through the intestinal membranes and the first-pass hepatic metabolism (degradation process within the liver) and in the end, to be subjected to the enzymatic degradation process within the blood. In order to control the degradation rate of the drug, it is necessary to create systems with a constant delivery rate and an approximately constant concentration of the drug in the plasma (to fall between the minimum effective concentration and the minimum toxic concentration) [18].

The stomach is an unfriendly environment for many drugs, which can be destroyed by its acidic pH (1.5–5, for fasted or fed conditions, see Table 1) or by the digestive enzymes (proteases, amylases, etc.). On the other hand, quite a lot of drugs can seriously injure the stomach, causing irritation or ulceration. Therefore, it is recommended that the drugs be used after their previous coating with pH-sensitive polymeric layers, which allow a release of the drug only at the pH corresponding to the small intestine, to protect both the drug and the stomach [19].

Gastro-retentive drug delivery systems (GRDDS) are new systems conceived to resist to the unfriendly environment within the stomach and to release the drugs in a sustained and prolonged manner in the upper part of the GIT. In this category are included the floating drug-delivery systems, which once reached the stomach, and float over the gastric fluids for an extended period of time, due to their much lower bulk density than of gastric fluids. These systems have the possibility to release the drug slowly, at a controllable rate, thus, increasing gastric retention time, and in the end are removed from the stomach. A possibility to increase the residence time of these systems is to produce systems as small particles, to make their ingestion easier, which after swallowing can increase their size when reach the gastric fluid [20].

The colon is part of the lower gastrointestinal tract (GIT) with a transit time of 20–30 h and a higher receptivity of its tissue to the absorption of drugs. The administration of colon drugs may be done in two ways, oral or rectal. Oral administration of compounds based on stimuli-sensitive polymers is taken into account especially for this region, due to the changes of pH throughout different regions within GIT or of the existence of microbial enzymes. This allows the use of both, polymeric carriers (for various drugs, peptides or proteins), and pH-sensitive hydrogels [21]. The colonic region has been shown to be more suitable for the administration of peptides and proteins than the small intestine. Localized drug delivery can be achieved in the colon region, but this is only possible if the drug is protected from unfriendly upper GIT environment. Drug formulations used in the colon region are extremely useful in the treatment of colon diseases, such as colonic infections by parasites, viruses or bacteria, colorectal cancer, or inflammatory bowel disease (IBD, ulcerative colitis or Crohn’s disease). These formulations have the advantage of local release of the required drug concentration, while reducing side effects due to release in the upper GIT or worthless systemic absorption [16]. Related to the rectal administration, such as suppositories and enemas, these are not very efficient due to great fluctuation in their distribution [16].

Difficulties associated with parenteral or oral delivery have led to the research of alternative pathways, such as ocular, nasal, pulmonary, vaginal, rectal, and transdermal [22].

### 2.5. Vaginal and Rectal Drug Delivery

Local and systemic treatments of women can be performed vaginally, a useful way both for health problems of the female reproductive system and as a general way of drugs administration (microbicides, antimicrobials, sex hormones) [12]. This aspect is possible due to the fact that the vagina has an extensive surface, a rich vascularity and, more importantly, the first-pass metabolism is avoided. However, the period of drugs release into the vagina and their bioavailability are strongly influenced by the severe conditions within this region, so to prolong the release time, while increasing the bioavailability of drugs through the vaginal epithelium, it is necessary to design special formulations with specific requirements [23].

The rectal route is conventionally used for the local treatment of rectal diseases, such as hemorrhoids [1]. However, because the drugs absorbed in the lower part of the rectum enter directly in the systemic circulation, the rectal route can additionally be used for the administration of drugs suffering intense first-pass metabolism [12].

Nevertheless, there are some limitations related to the vaginal and rectal delivery routes, such as the discomfort of the patient caused by the administration mode, or the drugs leakage from the application site as they melt in the cavities and, as for the rectum, the drugs diffuse from suppositories and begin to migrate to the colon. These aspects affect the drugs’ bioavailability, especially in those suffering from extensive first-pass elimination [24].

One possibility to control these disadvantages is the use of a different means of delivery (with a syringe device) of the thermo-reversible mucoadhesive in-situ gels, thus increasing the residence time by reducing the leakage and consequently, improving the bioavailability and effectiveness of the drug [1]. For instance, antifungal, antiseptic, antibiotic or contraceptive drugs (simple or in combination with other drugs) can be delivered in the form of a gel, via vaginal or rectal routes [24].

### 2.6. Cancer Drug Delivery

Cancer treatment is mainly based on chemotherapy which consists in the use of various chemical compounds in order to destroy cancer cells [25]. Although chemotherapy is a treatment that generally has positive results, it is well known that it has a high systemic toxicity, due to the reduced bioavailability of anti-tumoral drugs and a small half-life of their release [26,27]. Therefore, out of the need to achieve the desired therapeutic effect, high doses of drugs administered with a high frequency and which do not have undesirable side effects in treated patients are required [26].

One way to deliver drugs directly to cancerous tissue, without spreading them elsewhere, would be a possibility to reduce or even avoid unwanted side effects. For this, both the reduced therapeutic activity, the insolubility and toxicity of antitumor formulations, as well as the aspects related to the accessibility and heterogeneity of tumoral sites, must be taken into account [28]. Thus, special delivery systems are needed to ensure a safe encapsulation of drugs throughout the period until it reaches the tumor, so that when it enters the tumor, the systems disassemble [25].

Materials that accurately meet these requirements are stimuli-sensitive hydrogels, such as thermo-sensitive hydrogels for localized administration, which are highly effective in controlling the release of drugs to the tumor site, thus reducing possible side effects [27].

## 3. Hydrogels as Potential Drug Delivery Systems

Hydrogels make an important contribution to the evolution of controlled drug delivery systems. In recent years, hydrogels have increasingly attracted the attention of researchers regarding the design and preparation of controlled drug-delivery systems, due to their special properties which certifies them for biomedical applications [4,29,30,31]. Their high swelling degree with the capacity to retain a high amount of liquid and soft consistency, make them similar to living tissues [12]. Hydrogels have a number of advantages which make them suitable for drug-delivery applications, such as (i) biocompatibility, (ii) the ability to design and control their properties, (iii) the capacity to encapsulate water-soluble compounds, and (iv) the possibility of sustained and local release of active ingredients [29].

Usually, the initial step is realized outside the body and consists of the incorporation of the drugs within hydrogels, and afterwards the hydrogel-drug complex is introduced into the body, directly at the affected site. However, the principal drawback of this type of polymeric system is represented by the implantation of the voluminous material within body, through surgery. Therefore, in recent years, there has been increasing interest in the preparation of formulations that are in a solution state outside the body, but which, once injected inside the body, turn into a gel [32].

### 3.1. Properties of Hydrogels

**Figure 1:**
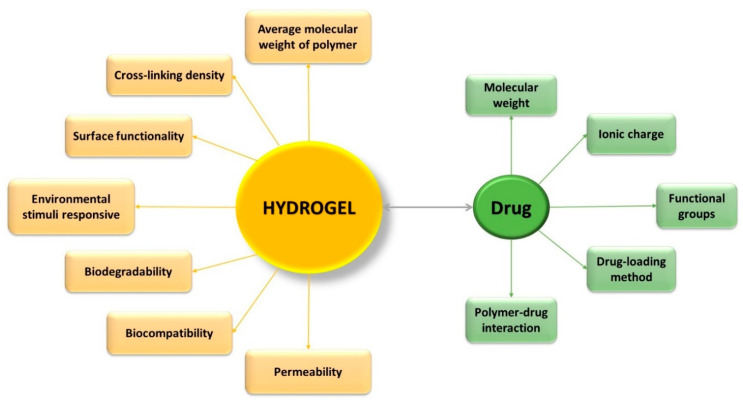
Characteristics which influence the effectiveness of controlled drug-delivery process.

Hydrogels have gained special interest for applications such as drug administration due to their unique physical properties. Their potential to work as a drug delivery system is a combination of various factors, such as (Figure 1) [32,33]: *volume fraction of polymer*—influences the amount of absorbed fluid by the hydrogel, and the *crosslinking degree*—have influence on the pore dimensions, thus directly on the structure of the hydrogel network, which can be correlated with the mechanical properties of the hydrogel, with its biodegradability, or with the processes of encapsulation/release of the drugs. Three of the most important parameters that characterize the structure of hydrogels are: *morphology*—their porous structure; *swelling degree*—which has a major influence on the mechanism of drug release from the polymer network; and *elasticity*—influence the mechanical properties of the network [9].

The drugs are released from the polymer network only through a diffusion mechanism and in this sense the type of porous structure of hydrogels is particularly important [34]. Depending on the pore size within the three-dimensional network of hydrogels, they can be classified as follows [11]:

- *macroporous*—average pore size: 0.1–1 μm—drug release: mechanism that depends on the matrix porosity and diffusion coefficient of the drug;
- *microporous*—average pore size: 100–1000 Å—drug release: molecular diffusion and convection;
- *non-porous*—average pore size: 10–100 Å—drug release: diffusion mechanism.

Researchers have designed and optimized the physical and chemical properties of the hydrogels for specific applications, such as sustained-release applications (permeability), pulsatile-release applications (environmental responsive nature), bioresorbable applications (biodegradability), and targeted release and bioadhesion applications (surface biorecognition sites) [35].

Hydrogels intended for use in medicinal products must be non-toxic and have properties such as biocompatibility, biodegradability and adequate physical and mechanical integrity [9,36].

*Biocompatibility* is sustained both by the high content of water within the hydrogel and by the similarities between the properties of hydrogels and those of the extracellular matrix [32]. The *toxicity* of hydrogels is mainly related to the toxicity of the unreacted components of hydrogels, such as monomers, oligomers, initiators, etc. To reduce the toxic effects of the hydrogels, the following must be taken into account: (i) to avoid the use of initiators, (ii) to remove the impurities by a powerful washing, or (iii) to use reactions with high rates of conversion, in order to eliminate the unreacted monomers and by-products [1]. *Biodegradability* is not always necessary for hydrogel formulations. This depends on the location where the drug delivery device is used. Therefore, it is not necessary for oral and transdermal drug administration, while it is absolutely necessary when hydrogels are used to various parts inside the body, in order to avoid unpleasant reactions of the human body to foreign bodies in the organism and even their surgical removal [37,38]. The hydrogels’ biodegradability can be achieved by different ways: as chemical and enzymatic oxidation, hydrolytic degradation, enzymatic degradation or thermal and mechanical degradation [32].

In the applications where biodegradability is not absolutely necessary, it is even more important to keep the integrity of the hydrogel, due to situations where the drugs need to be protected from the severe conditions within the body, until the drugs can be delivered to the target site [1]. The *hydrogel strength* can be improved either by incorporating of nanoparticles, or through raising the cross-linking degree. Nevertheless, the degree of cross-linking cannot be increased too much, due to the fact that a higher cross-linking degree induces a loss of elasticity, with the hydrogel becoming brittle. In addition, the elasticity is an important property of the gel because it gives flexibility to the 3D network, contributing to the circulation of the incorporated therapeutic agent within the polymeric network. That is why it is necessary to achieve a compromise between the mechanical strength and the elasticity of the hydrogels for a proper application of these [39,40].

### 3.2. Hydrogels Delivery Systems

The delivery systems based on hydrogels used for controlled drug release can be classified into reservoir and matrix devices [40,41,42,43,44].

#### 3.2.1. Reservoir System

**Figure 2:**
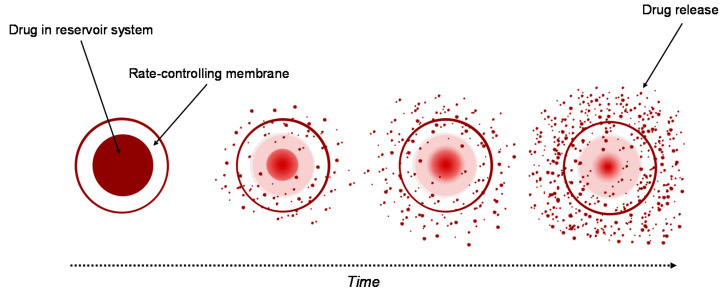
Drug delivery from reservoir device.

Currently, the reservoir-based system is the most widely used for controlled drug delivery. In these types of system, the drug core is covered all around by a hydrogel membrane (Figure 2). The membrane thickness and the properties of the entrapped drug, like solubility, molecular weight and size of particle, control the release rate of the drug [43,44]. Upon contact with water, it diffuses through the hydrogel membrane and dissolves the drug until it reaches saturation solubility (Cs). As the drug diffuses through the membrane to the external medium, the drug concentration in the reservoir decreases below Cs, so that new amounts of the solid drug present in the core dissolve and the concentration Cs are restored. This ensures a constant rate of the release of the drug from a reservoir-based system that follows zero-order kinetics if the solid drug is still present in the core [40].

#### 3.2.2. Matrix System

**Figure 3:**
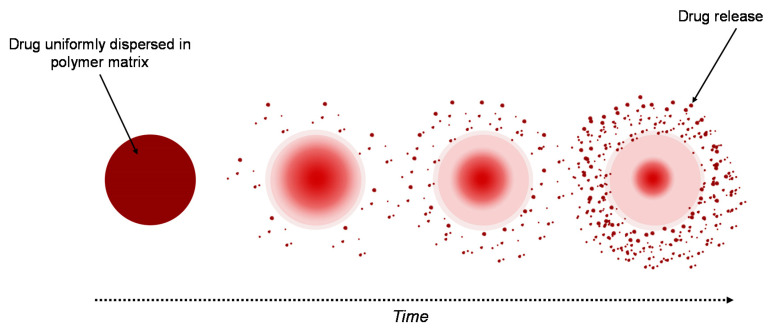
Drug delivery from a matrix system.

A similar system to the reservoir-based system is the matrix-type delivery system, with the observation that in this case, the drug is uniformly distributed as a solid into a hydrogel matrix (Figure 3) [40,42]. A matrix is composed of one or more drugs along with a hydrophilic polymer, as a gelling agent [45]. In both types of delivery system, drugs are retained within the polymer matrices, but are non-covalent [43].

The drug release strongly depends on the matrix’s properties. When the system is placed into aqueous medium, water diffuses into the matrix hydrating it from the surface to the core. Three important processes control the release of drugs, these being: (i) the process of diffusion of water into the matrix, (ii) the process of dissolution of the drug, and (iii) the process of diffusion of the drug from the system. The polymer–drug interactions have an important role in the release process of the drug, in this case. Furthermore, the thickness of the hydrated matrix is included as the diffusional path length of the drug [40]. A sustained-release matrix-type drug-delivery system has an important role in increasing the therapeutic effectiveness of the drugs by controlled and sustained release, and by selecting the desired site. For a specific period of time, a constant released level of the drug is maintained, so that the adverse effects are avoided [12].

### 3.3. Hydrogel Drug-Release Mechanisms

The main criterion for classifying the controlled drug-release processes is the mechanism which leads the release of the active pharmaceutical ingredient (API). The release mechanism is a result of three main processes: (i) drug diffusion, (ii) matrix swelling, and (iii) chemical reactivity of the drug/matrix [37]. Various mathematical models were developed in order to estimate the drug release as a function of time, which are based on the limiting rate of the release process [9,13,33]. The mechanisms for the drug release and the corresponding hydrogel-type delivery systems can be classified as following [46,47,48,49,50]:

− *diffusion-controlled*—matrix and reservoir systems;
− *swelling-controlled*—systems that are osmotically controlled and are activated with different solvents;
− *chemically controlled*—systems that can be biodegraded or eroded and pendant chain systems.

**Figure 4:**
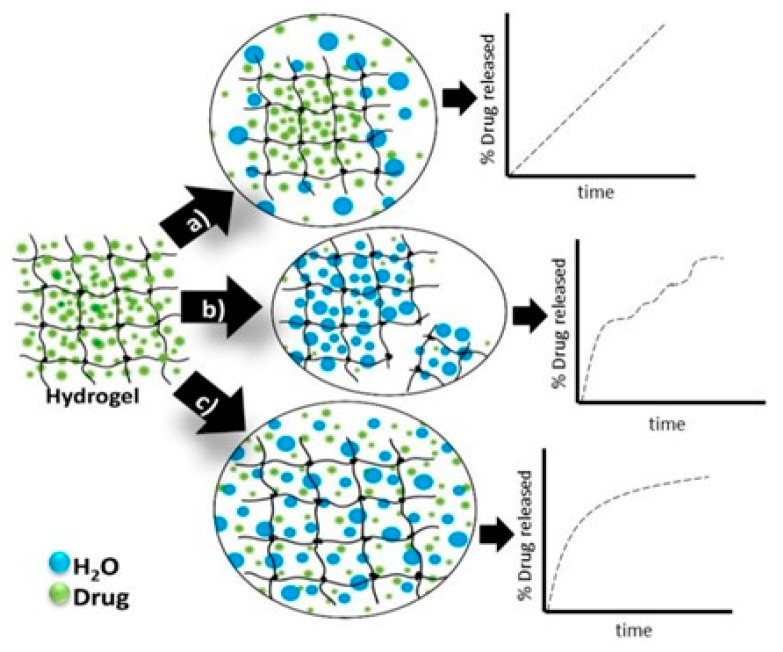
Hydrogels drug-release mechanisms and their respective kinetic profiles: (**a**) the case when the governing mechanism is given by the drug diffusion; (**b**) when it is imposed by the degradation of the polymeric matrix; (**c**) when the hydrogel swelling governs the process [51].

In Figure 4 are presented the schematic view of the drug release mechanism from hydrogels [51].

Passive diffusion is the most common release mechanism. In this mechanism, depending on the mesh size of the matrix, the biotherapeutic molecules entrapped within the matrix can diffuse freely. In the case of systems in which the release of active principles is based on an erosion-controlled mechanism, there is a close dependence between the rate of drug release and the rate of erosion. In the latter case of mechanism, chemically controlled release is based on a series of chemical reactions that produce either the degradation of the polymer in the hydrogel matrix by hydrolytic or enzymatic reactions or reactions of cleavage of the polymer–drug bonds [27]. 

#### 3.3.1. Diffusion-Controlled Drug Mechanism

As shown above, the diffusion-controlled release is the most common mechanism of drug release from hydrogels and it is used by reservoir or matrix devices [13]. Reservoir-type delivery systems offers a constant and time-independent release of the drug, while the matrix system is one time-dependent drug release system and its working depends on the size of the open space or macromolecular mesh. For a time-dependent system, the initial release rate is variable and is proportional to the square root of time [52].

**Figure 5:**
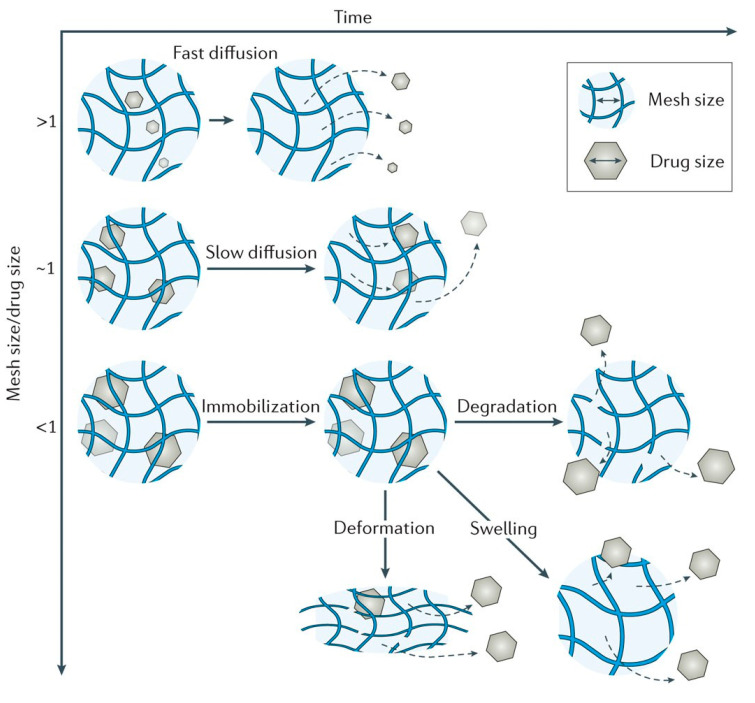
Mesh size mediates drug diffusion [53].

Hydrogels are in fact cross-linked polymer networks with open spaces between polymer chains, called meshes, which permit the diffusion for liquids and small solutes. The most important feature is the mesh size because it influences the steric interactions between the network and the drug, and ultimately determines how the drug is released from the hydrogel. Depending on the ratio in which the mesh sizes (Rmesh) and the size of the drug molecules (Rdrug) are found, the following three situations can be identified (Figure 5) [53]:

- *Rmesh/Rdrug > 1*, mesh size is larger than the drug molecules: the whole release process is controlled by diffusion. It is the case of small drug molecules which diffuse freely through the network, and their migration is not dependent on the mesh size;
- *Rmesh/Rdrug ~ 1*, mesh size reaches the drug size: the steric hindrance dominates the drug diffusion. The resulting effect is a slow drug diffusion, which is reflected by a slow and extended-release;
- *Rmesh/Rdrug < 1*, mesh size is very small and/or drug molecules are too large. The effect of steric hindrance causes a blockage of the drug within the network, until there is a degradation of the network or an increase in mesh size by swelling or deformation.

#### 3.3.2. Swelling-Controlled Drug-Release Mechanism

Another possibility to release enclosed drugs is to control the swelling process of hydrogels. Swelling-controlled drug release could occur when the rate of drug diffusion is faster than the rate of hydrogel swelling, the higher the rate of hydrogel swelling, the higher the rate of drug release. Thus, the rate and ability of hydrogels to absorb water and the thickness of polymeric gels are important factors in swelling-controlled delivery systems [13].

A shortcoming of controlled swelling systems is the too slow response of macroscopic hydrogels due to the slow diffusion of water. To obtain a faster reaction, the diffusion length can be reduced either by decreasing the size of the hydrogel or by designing a system of interconnected macropores into the volume of the hydrogel [53].

#### 3.3.3. Chemically-Controlled Drug-Release Mechanism

Chemically controlled delivery systems can release the encapsulated drug by breaking the polymer chains as an effect of surface or bulk erosion [13]. In erodible drug delivery systems, drug release is controlled by either the dissolution or degradation process. Depending on the limiting process, the drug is released through a different mechanism: if erosion is the slower process then the rate of release will be controlled by diffusion; and if drug diffusion is a slow process, then degradation or erosion controls the drug release [9].

Erosion processes of hydrogels can take place in bulk or on the surface. Bulk erosion is the most common in the case of hydrogels because their network is permeable to the main actors of the degradation process, water and enzymes. Conversely, if the rate of cleavage of the bonds is higher than the rate of diffusion of water inside the hydrogel then erosion occurs at the surface [53].

## 4. Cellulose-Based Hydrogels in Drug Delivery

### 4.1. Cellulose and Cellulose Derivative-Based Hydrogels Properties

Cellulose is the most known biodegradable polymer, being the main component of all vegetable fibers, with properties ranging from low cost and biocompatibility to high mechanical and thermal stability, which makes it a very promising and attractive polymer for various applications [54,55,56,57]. Its only drawback is its low solubility, which restricts its use especially in the biomedical and pharmaceutical fields, but this disadvantage can be overcome by obtaining cellulose derivatives by various chemical modification procedures, such as esterification, etherification, or oxidation [29,54,58,59]. Cellulose and cellulose derivatives are used in the pharmaceutical industries as “excipients” to control the rate of drug release and to reach the correct drug concentration [60].

Nanocellulose (NC) has a number of properties essential for biomedical applications, such as biocompatibility, non-toxicity, wound-healing properties, and antimicrobial effects, but also the high binding potential by electrostatic adsorption on tissues due to the multitude of OH groups available and negative interface charge [61,62].

NC can be classified into two main categories, cellulose nanocrystals (CNC) and cellulose nanofibrils (CNF). CNC is a highly crystalline, needle-like form of nanocellulose, which has been obtained from wood pulp, cotton, cellulosic agricultural residues, or microcrystalline cellulose (MCC) by acid hydrolysis [63]. CNC has a number of advantages over cellulose fibers, such as nanoscale size, high strength and specific modulus, high specific surface area and special optical properties which give it wide range of applications.

CNF consists of nanosized cellulose fibers composed of long and flexible cellulose chains, arranged in a structure governed by hydrogen bonds [62]. CNF is characterized by a recognized cytocompatibility and a high tolerogenic potential. These are the main arguments supporting the use of CNF in various clinical applications [64]. However, CNFs have two main disadvantages, closely related to its physical properties. The first is the high capacity of nanofibrils to interact and bind through a large number of hydrogen bridges, which alters the desired structure in drying processes. The second disadvantage is the high hydrophilicity of cellulose, which prevents the use of this material in a number of applications. Therefore, by adjusting the loading density of the fibers, by properly choosing the conditions of the swelling environment and the working methodology, hydrogels with a controllable degree of swelling, high porosity and high specific surface area can be obtained [64].

In recent years, bacterial cellulose (BC) produced by species such as *Acetobacter xylinum* has been increasingly used in biomedical applications. This type of cellulose has a microfibrillar structure, nanostructured, which causes higher water retention and a high degree of biocompatibility [65]. BC has demonstrated superior properties to cellulose obtained from plants and satisfies essential requirements as a material for biomedical applications up to the ease of sterilization [66].

Cellulose-based hydrogels have properties that recommend them for various biomedical applications, such as targeted delivery of drugs and smart sensors, or hydrogels multi-receptive, injectable and self-healing [67,68].

**Figure 6:**
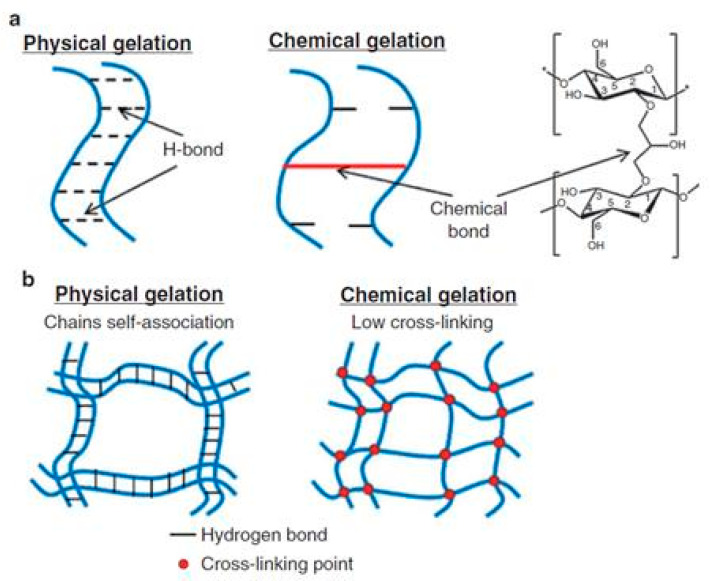
(**a**) A sketch of network formation in cellulose solutions: physical gelation via self-association of chains and chemical cross-linking; (**b**) a schematic presentation of the structures of physical and chemical cellulose gels [70].

Cellulose-based hydrogels can be easily prepared either by interactions of physical nature (mechanical chain entanglements, van der Waals interaction, hydrogen bridges, hydrophobic or electronic associations) or by chemical cross-linking (with crosslinking agents) [69]. Physical gelation involves the self-association of cellulose chains due to preferential cellulose–cellulose interactions and not cellulose-solvent, often accompanied by a micro-phase separation. Chemical gelation disrupts the self-association and packing of cellulose chains leading to swollen transparent coagulated cellulose hydrogels with a more homogeneous morphology and a more porous structure, lower crystallinity, higher swelling degrees and a higher affinity for water vapor adsorption (Figure 6) [70].

The two different processes of preparation (physically and chemically) lead to hydrogels with different structures and degrees of swelling that are reflected in the ability to load and release drugs. Chemically crosslinked hydrogels can be loaded with greater amounts of drug that they release faster compared to hydrogels resulting from physical self-association [71]. Cellulose hydrogels with controlled morphology and porosity may be prepared by using optimum proportion of amounts of cellulose and the cross-linker.

Cellulose derivatives, depending on the type of the functional groups, are able to form either physical hydrogels or crosslinked chemical hydrogels. In physically associated hydrogels the chains of cellulose derivatives are aggregated by hydrogen bonds, ionic interactions or even hydrophobic forces. Various crosslinking agents and catalysts are used for the chemical crosslinking of cellulose derivatives. The most used crosslinking agents are dialdehydes, acetals, polycarboxylic acids, epichlorohydrin and polyepichlorohydrin [69].

Cellulose ethers are among the most widely used cellulose derivatives in obtaining drug formulations in the pharmaceutical industry. They are mainly used as excipients and may include methylcellulose (MC), carboxymethylcellulose (CMC), ethyl cellulose (EC), hydroxyethyl cellulose (HEC), hydroxypropyl cellulose (HPC) and hydroxypropyl methylcellulose (HPMC) [38]. Cellulose ethers are hydrophilic compounds, which can form gels in the presence of a high amount of water. Moreover, hydrogels in cellulose ethers are biodegradable, biocompatible and have adequate characteristics for a targeted and sustained release of the drug over a long period of time, having the ability to deliver the drug even in the presence of a specific environmental stimulus [3].

CMC is the most commonly used cellulose ether in drug administration and other biomedical applications, due to it essential characteristics such as hydrophilicity, bioadhesivity, pH sensitivity and non-toxicity. The presence of CMC in controlled drug-delivery systems can hinder crystallization or degradation of the drug and can enhance the frequency of drug release by increasing the rate of drug diffusion or the rate of polymer erosion/degradation [72]. 

Ethyl cellulose (EC) is extensively used in controlled release formulations due to its hydrophobic nature. EC is mostly used in drug-release formulations especially for colonic diseases, as a coating agent together with a biodegradable polymer. Also, EC can be used to improve the mechanical properties of the films prepared from various polysaccharides, which show increased fragility due to its high swelling capacity in water.

HPMC is biocompatible, has hydration and gel forming properties and has global regulatory acceptance to be used in the preparation of various pharmaceutical formulations. HPMC is usually used to extend the release time of drugs. For example, was used in the formulation of hydrodynamically balanced systems for the specific administration of the drugs to the stomach [73], or have been tested in various oral administration systems due to its mucoadhesive properties [66]. Furthermore, HPMC can be used in the preparation of drug-delivery systems in the form of reservoir-type devices coated with a barrier layer. The time for drug release from a reservoir device is determined by the coating thickness [74].

Hydroxypropyl cellulose (HPC) is a biodegradable and biocompatible polymer, with self-repairing abilities, shape memory, and a unique hydrophilic/hydrophobic change, as a function of external stimuli such as pH, temperature, pressure, light, magnetic or electric fields. For the controlled delivery of hydrophilic drugs, HPC was used in preparation of thermo-responsive hydrogels [75].

Cellulose esters are synthesized by esterification of hydroxyl groups with various organic acids in the presence of a strong acid as a catalyst. In the pharmaceutical area, the most used esters are cellulose acetate (CA), cellulose nitrate, cellulose acetate phthalate (CAS), hydroxypropyl methylcellulose phthalate (HPMCP), and hydroxypropyl methylcellulose acetate succinate (HPMC-AS). These esters are insoluble in water and have good characteristics for film-forming, useful properties for standard coatings [54]. Moreover, cellulose esters are non-toxic, stable, are not absorbed from GIT and have the relatively high permeability to water, properties which are essential in drug-delivery applications [19].

Hydrogels based on cellulose derivatives have important applications as drug delivery systems (DDS) and are used in order to improve the controlled release of drugs, as a function of external stimuli, such as body temperature and variable pH ranges in different parts of the body [3].

**Figure 7:**
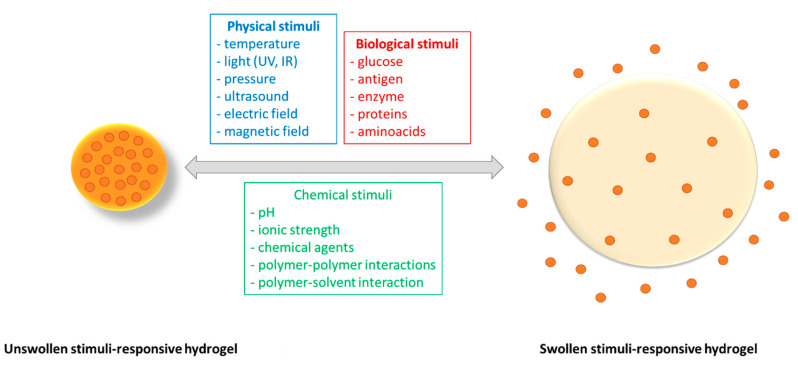
The schematic representation of stimuli-responsive hydrogels.

Stimuli-responsive cellulose hydrogels undergo mighty modifications in their network structure, swelling degree, permeability, etc. as a reaction to external stimuli, and are capable of showing switchable sol-gel transition on the application of different external stimuli. Generally, the hydrogels are divided into physical, chemical or biological responsive hydrogels, (Figure 7) as they react to [76,77]: (i) physical stimuli: temperature, pressure, light, electric or magnetic fields, (ii) chemical stimuli: pH, chemical agents, ionic factors, etc., or (iii) biological stimuli: proteins, enzyme, glucose, etc.

Smart hydrogels have morphological and functional characteristics that change in the presence of various external stimuli, essential properties for the applications in the field of drug delivery systems. These hydrogels are designed in order to obtain systems with controlled and sustained drug release and with lowest side effects [78,79,80,81,82,83].

Various research studies have been conducted to demonstrate the effectiveness of cellulose-derived hydrogels in the controlled and sustained release of drugs, and some of them are presented in Table 2.

### 4.2. Cellulose-Based Hydrogels for Oral Administration

#### 4.2.1. Oral Cavity Delivery

In recent decades, for the treatment of oral diseases, such as periodontitis, viral or fungal infections, treatments with the possibility of releasing drugs directly into the oral cavity have been used. In this case, due to the abundant local salivary flow which bathes the oral cavity mucosa, it is necessary to use hydrogels with long-term adhesion capacity to achieve local drug delivery. 

CMC is a polymer with bioadhesive properties, a characteristic that allows it to adhere to various biological surfaces. With these properties, CMC can be successfully used in the administration of drugs such as miconazole nitrate or lidocaine hydrochloride, in transmucosal applications (miconazole nitrate gave reasonable buccoadhesion time between 2.45–3.65 h; on the other hand, lidocaine hydrochloride remained well above the minimum effective concentration of 4–10 mg/mL in saliva for a period of 6 h) [72].

Hydroxypropyl cellulose (HPC) hydrogels are also suitable for preparation of bioadhesive hydrogel systems, by using it in combination with poly(acrylic acid) (PAA) and a lactose non-adhesive backing layer. These systems are commercially available as Aftach and are used in the treatment of aphthous ulcers, by releasing triamcinolone acetonide [98].

The oral cavity is also a favorite route for delivering drugs to various regions of the GIT, like the stomach, small intestine, or large intestine.

The bicomponent hydrogels based on cellulose (C) and chondroitin sulphate (CS) were prepared by chemically cross-linking and were evaluated in vitro and in vivo as drug (theophylline) release systems. By using near-infrared chemical imaging (NIR-CI) a uniform distribution of the theophylline in the C/CS sulfate hydrogels was proved. Regarding the in vitro release (in acidic medium), it was shown that an increase in the CS content causes a decrease in the percentage of theophylline release, such as: 95% (C), 78% (80/20 C/CS), 58% (50/50 C/CS). Also, this rise determined a decrease of the half release time values, which vary from 14 min for C hydrogels to 60 min for 50/50 C/CS composition. In vivo release (the drug was administered orally to rats as suspensions in 0.5% wt CMC) indicated a higher biocompatibility of the drug incorporated in the polymeric matrix than of the pure drug and a prolonged release of theophylline of up to 50 h for the matrix containing 50% C and 50% CS [99]. Furthermore, the C/CS hydrogels have good biocompatibility demonstrated by the hemolysis (plasma hemoglobin) technique [100].

When passing from the stomach to the intestine there are large variations in pH, which allows cellulose-based hydrogels, in particular based on cellulose derivatives, a controlled and prolonged release of drugs. Hydrogels based on HEC, CMC, hyaluronic acid (HA), sodium alginate, xanthan, carbopol, as well as polycarbophil and porcine mucus were ex vivo studied, in order to establish the resistance to removal by the flow of artificial saliva. An increase of the residence time was observed as follows: carbopol < hydroxyethyl cellulose, hyaluronic acid, alginate, polycarbophil < carboxymethyl cellulose < xanthan [101].

#### 4.2.2. Stomach-Specific Drug Delivery

In order to confer a protection against the strong acidic environment from the stomach, for the protein drug release, hydrogels made of sodium acrylate and CMC were used, which in addition allowed a controlled release of the drug into the intestinal fluid [72].

**Figure 8:**
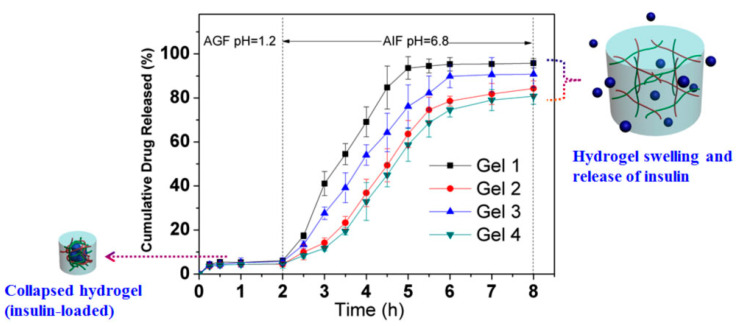
In vitro release profiles of insulin from hydrogels after incubation of insulin-loaded gels in artificial gastric fluid (AGF) for 2 h at 37 °C, followed by incubation in AIF for another 6 h at 37 °C [102].

Traditional insulin administration is performed by subcutaneous injection and long-term repeated injections determine a reduced acceptance of treatment by the patient. Thus, to avoid these inconvenient pH-responsive hydrogels made from acrylate-grafted CMC and PAA were developed for oral delivery of insulin, in order to increase the comfort of the patient [102]. Due to different swelling behaviors as a function of pH, the hydrogels exhibited different enzymatic degradation (Figure 8).

Thus, the enzymatic degradation in artificial gastric fluid (AGF, pH 1.2) with pepsin was stopped, while in artificial intestinal fluid (AIF, pH 6.8) with pancreatin it was increased. There was also an increase in the percentage of insulin released from hydrogels, in the case of the environment that mimics the small intestine. In AGF, less than 10% of the loaded insulin was released after 2 h. In contrast, in AIF the release rates were enhanced markedly, after 6 h reaching values between 95.8% and 80.8% depending on the hydrogels’ composition. In the in vivo experiment, oral administration of insulin-containing hydrogels in the treatment of diabetic rats, resulted in decreased glycemic levels and was shown to be much more effective (more than 10-fold) than pure insulin solution [102].

A pH- and temperature-dependent drug-delivery system has been obtained by mixing MC and alginate. The drug is loaded in the mixed polymeric solutions, at room temperature, and the obtained system turns into a gel with the increase of temperature upon entering the human body, due to the thermo-gelling properties of methylcellulose and pH sensitivity of alginate in contact with a medium at low pH. Thus, in simulated gastric fluid (SGF), the hydrogel delivery system was able to release bovine serum albumin (BSA) at a lower rate compared to the neutral environment of simulated intestinal fluid (SIF) [103].

Another study evaluates HPMC hydrogel in combination with chitosan to obtain stomach-specific drug delivery carriers for moxifloxacin HCl (MX) as a model drug, used as a broad-spectrum antibacterial agent [73]. To delay the release of the drug from hydrogels, HPMCs with high degrees of polymerization were used. Upon contact with the dissolution medium, the HPMC matrices swell strongly and form a thick gelatinous layer around the matrix, which has eroded at the same time (the hydrogels formulation exhibited 20%, 57% and 74% MX release within 1 h, 6 h and 8 h respectively, after exposure to the dissolution medium). Experimental results support the use of HPMC in combination with chitosan as a potential carrier material for sustained release systems of hydrophilic drugs as a therapy for stomach dysfunction, at high doses and with the specificity of absorption in the upper part of GIT.

#### 4.2.3. Colon-Specific Drug Delivery

Due to the fact that in the region of the colon there are high concentrations of polysaccharide enzymes, hydrogels based on polysaccharides or polyesters were designed and realized, specific to this area. The release of the drugs from these hydrogels was proposed to be done either at changes in pH, or under the action of enzymatic degradation [21,34]. 

Hydrogels prepared from CMC and acrylic acid (AA) by γ-radiation induced copolymerization and crosslinking, where used and evaluated as drug carriers for colon. Swelling kinetics studies show that the hydrogel has a Fickian diffusion in the stomach medium (pH 1), and a non-Fickian diffusion in the intestine medium (pH 7), while the drug release (theophylline) showed dependence on the pH (insignificant at pH 1 and very fast at pH 7), on the copolymer composition (the increase of CMC content from 25 to 50 wt% increases the amount of the drug released up to about 4 times), and also on the irradiation dose during hydrogel preparation (the drug released amount increase with irradiation amplification, e.g., from 200 mg theophylline/g dry gel at 10 kGy to 800 mg theophylline/g dry gel at 30 kGy). All these properties recommend the hydrogels as a site-specific drug carrier [7]. 

Other polymer blends have also been investigated as controlled drug-delivery systems for the colon and have been shown to be suitable for use in the treatment of colon diseases. These were made from ethyl cellulose (EC), starch, amylose, pectin and calcium pectinate [21].

**Figure 9:**
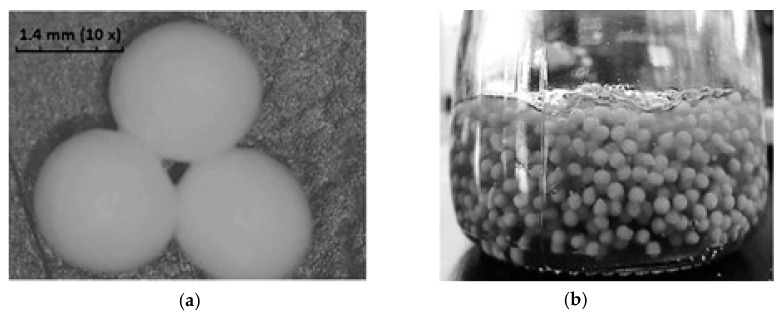
(**a**) Carbamazepine (CBZ)-alginate beads, (**b**) CBZ-gel prepared at a 1:1 ratio of beads to iota carrageenan gel [104].

Microparticles from ethyl cellulose were prepared by a solvent evaporation method, in order to develop a sustained release formulation of carbamazepine (CBZ), suitable for dysphagic patients, especially for children below six years old [104]. The entire controlled drug-delivery (CBZ) system was accomplished by encapsulating the microparticles in alginate beads, which were then suspended in an iota-carrageenan gel (Figure 9).

The microparticles had a monodisperse distribution, with a particle size of 135 ± 0.61 μm and encapsulation efficiency of 84 ± 3.98%, while the beads’ average size was 1.4 ± 0.05 mm and they were monodispersed, with a sphericity factor less than 0.05. Following this study, it could be concluded that the system has different advantages in terms of dosing flexibility (suspension form) and controlled release profile (tablet form).

New bacterial cellulose-g-poly(acrylic acid-*co*-acrylamide) (BC-g-poly(AA-*co*-AM)) hydrogels were prepared by a microwave irradiation technique, as a controlled-drug delivery system (theophylline) for the lower GIT. Swelling studies have shown that hydrogels have been sensitive to changes in temperature and pH. Although initially, at pH 2, the hydrogels showed a low initial swelling ratio, as the pH increased, mainly due to AA ionization, the swelling ratio continuously increased reaching a maximum at pH 7. Finally, the in-vitro drug release profile of the hydrogels showed a lower level of drug release in SGF than in SIF because of the significant pH-sensitivity of these hydrogels (in SGF, the hydrogels showed a cumulative release of almost 12% after 2 h, whereas the total release from the hydrogels reached up to 90% in SIF). The hydrogels showed also a reduced swelling at body temperature suggesting that they can be applied for temperature-controlled delivery [105].

Stimuli-responsive BC grafted with polyacrylic acid (BC-g-PAA) hydrogels were prepared by electron-beam (EB) irradiation and demonstrated their potential for site-specific delivery of protein-based drugs in the intestine (bovine serum albumin—BSA). Both structural integrity and protein bioactivity have been preserved by these hydrogels. In addition, the excellent mucoadhesive potential demonstrated by hydrogels can increase residence time and improve drug absorption, as protein penetration studies have shown. The BC-g-P(AA) hydrogels exhibited better control on the BSA release in SGF (<10%) and higher maximum release (~90%) in the first 2 h in SIF; among the hydrogels, those with 80% BC have the highest cumulative release (90%), likely due to its greater pore size and swelling. Related to the in vivo applications, these hydrogels proved to be suitable due to excellent results related to the cytotoxicity and acute oral toxicity. The cell viability was well above 90%, most likely due to the presence of BC that has been reported to enhance cell proliferation and cell attachment [106].

**Figure 10:**
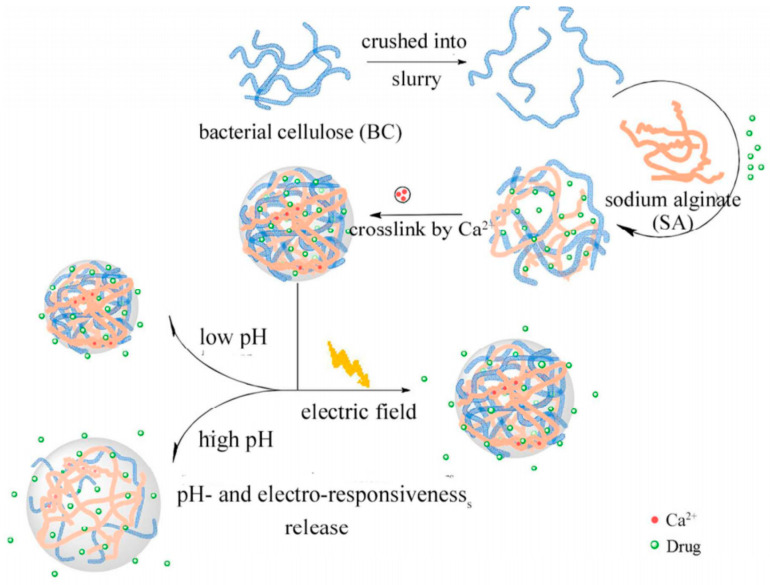
Releasing process for the drug loaded bacterial cellulose nanofiber and sodium alginate (nf-BC/SA) hybrid hydrogels under different pH and in various electric field strengths [107].

Hydrogels based on bacterial cellulose nanofiber and sodium alginate (nf-BC/SA) were prepared and these were investigated as stimulus-responsive drug (ibuprofen, IBU) release systems. The introduction of BC nanofibers within the hydrogels permitted more stable microstructures to be obtained and enhanced their electro-responsive properties. Related to the swelling ratio of the nf-BC/SA hydrogels, this was 8 times less in acidic medium of pH = 1.5 and of 13 times more in alkaline medium (pH of 11.8), which could be explained by the ionization of SA at a high pH. Moreover, at a voltage of 0–0.5 V, an increase of the swelling degree from 8 to 14 times was recorded. In vitro tests of nf-BC/SA hybrid hydrogels proved the dependence of controlled-release of the drug on the pH value (quickly in neutral or alkaline media and slow in acidic media) and on an electric stimulus, when compared with the passive release (during the first 2 h, the drug released about 10% at 0 V due to the lower swelling of hydrogels, as it reached 23% and 38% at 0.3 V and 0.5 V, respectively; after 8 h, the amount of drug released at 0.5 V was twice that of 0 V) (Figure 10) [107].

Recently, metal-organic frameworks (MOFs) demonstrated superior biocompatibility, biodegradability, and loading capacity, properties that recommend them as potential candidates for controlled drug release.

Javanbakht and coworkers [108] synthesized a Cu-based metal-organic framework (Cu-MOF) for encapsulation of a model drug, ibuprofen (IBU), a non-steroidal drug with anti-inflammatory and analgesic properties. In order to reduce the side effect of this drug, such as the irritation of the gastrointestinal tract (GIT), this was encapsulated into Cu-MOFs and CMC/Cu-MOFs. Following the study, it was concluded that the CMC capsulated Cu-MOF@IBU nanocomposite hydrogel bead was the most adequate matrices for a controlled-release of IBU, due to the fact that this demonstrated a better protection of IBU against the stomach acidic and a high stability of drug release for a long period of time (the CuMOF@IBU exhibited a fast drug release at pH 1.2, 95% of the drug being released after 120 min; while for CMC/Cu-MOF@IBU, the amount of released drug was 70% after 480 min, in a controlled manner).

### 4.3. Cellulose-Based Hydrogels for Dermal and Transdermal Administration

Hydrogels are hydrophilic three-dimensional matrices which exhibit indispensable properties for the dermatological applications, such as the fact that they: (i) are transparent, (ii) greaseless, (iii) and thixotropic, (iv) have good bioadhesive properties and (iv) are suitable for the incorporation of drugs, active pharmaceutical ingredients, lipophilic compounds or different solids [8].

Among these, cellulose-based hydrogels have attracted special attention for their properties, as they: (i) are non-toxic compounds, (ii) biocompatible materials, (iii) have a strength which can be correlated with the cross-linking degree between the polymers, (iv) good mechanical properties, and (v) have a reversible viscosity, that can be influenced by modifications of external factors, as temperature, pH, etc. [3].

#### 4.3.1. Dermal Administration

The treatment of dermatological diseases, with different degrees of skin condition, such as: irritations, acne, atopic dermatitis, rosacea and allergies, can be achieved by using cellulose-based hydrogels that have incorporated specific dermal drugs. Model medicinal products incorporated into cellulose-based hydrogels which have previously been solubilized in emulsion or microemulsion systems include: (i) fluconazole (an antifungal agent) and (ii) diclofenac sodium or (iii) piroxicam (known as the name of non-steroidal anti-inflammatory drugs) [8]. 

To establish an appropriate formulation (emulsion, lipogel or microemulsion-based hydrogel) for the treatment of topical mycosis, Salerno and colleagues [109] studied the in vitro release of fluconazole and developed a system containing sodium CMC as a gelling agent, propylene glycol (PPG) as a solvent for drug and diethylene glycol monoethyl ether (DEGEE) as a penetration enhancer. The study showed that the microemulsion-based hydrogel formulation is very effective in terms of in vitro antifungal activity, thus the microemulsion-containing DEGEE resulted in being the most effective one, being as effective as the standard solution, e.g., <10 colony-forming units (cfu)/mL), demonstrating the formulation’s ability to release the full amount of drug, the effectiveness of penetrating pig skin, even four times greater than lipogels, and the ability to store the drug in skin layers.

In another study, hydrophilic gels based on HPMC, chitosan and Poloxamer 407 are used as gelling agents, PPG as a solvent and various penetration enhancing agents, in order to establish the controlled-release capacity of fluconazole from the network. HPMC-based hydrogels were found to release the highest drug concentration after 6 h, in the presence (66.66%) or absence of penetration enhancers (71.65%) [73].

HPMC-based hydrogel was also tested as a topical drug delivery system for diclofenac sodium and compared with Carbopol 934P and sodium alginate gel formulations, respectively. HPMC has been shown to form a homogeneous, stable, water-washable gel due to its solubility in water, which does not cause irritation and has good permeability in vitro [1].

Several methylcellulose (MC) hydrogels, CMC, HPMC, carbopols 934 and 940 or Pluronic F-127 have been evaluated to determine the optimal formulation required for the controlled release of piroxicam (an anti-inflammatory drug) [110]. In this regard, piroxicam was incorporated as a microemulsion. Other chemicals used as surfactants were Tween 80 and propylene glycol. The results of the in vitro study indicated that the hydrogels based on 3% MC and 3% HPMC, respectively, released the largest amounts of drug, 97% and 94%, respectively, after only 3 h of experiment. Taking into account both the rheological properties and the duration of use of the hydrogel, it has been established that the hydrogel based on HPMC is the most suitable for use in topical administration of piroxicam.

For the case of a herpes virus infection, the controlled release was studied of acyclovir (ACV), an antiviral drug, from hydrogels prepared from CMC, β-cyclodextrin (β-CD), acrylic acid (AA) and *N*,*N*′-methylene-bis-acrylamide (MBA). The classic way to administer acyclovir is both oral and intravenous, high doses that have many side effects. Therefore, it was sought to achieve a drug-controlled release system, with a higher load capacity, but which would provide the desired release profile. A solution in this regard was the use of β-CD and CMC, which allows hydrogels with good mechanical properties to be obtained, but also with an improved capacity to load and release the antiviral drug. ACV release increases with increasing concentration of β-CD from 1% to 4% and CMC from 8% to 12%. The hydrogel with optimum composition—30 g AA/100 g, 4 g β-CD/100 g, 12 g CMC/100 g, and 0.6 g MBA/100 g—showed a maximum cumulative drug release of 96.15% at pH 7.4 among all hydrogels [111].

Another way to use hydrogels is to treat severely healed wounds, burns, or other skin conditions. Thus, the hydrogels based on poly(vinyl alcohol) (PVA) and oxidized cellulose (OxC) were prepared by the freezing/thawing method and were tested as controlled L-arginine release systems (e.g., after the first 30 min, the amount of L-arginine released was 66.92% by the hydrogel with 5% OxC and 85.8% for the one with 20% OxC; after 3 h, the percentage increased to 83.68% and 98.52%, respectively) [112].

#### 4.3.2. Transdermal Administration

A promising alternative to the conventional drug-delivery methods, such as oral administration and injection, is the transdermal drug delivery, which is considered a proper method of systemic delivery of drugs.

A transdermal film developed from cellulose nanofibrils (CNF) and chitosan was investigated for the delivery of ketorolac tromethamine (KT), when it was demonstrated that the incorporation of CNFs into chitosan permitted a sustained drug release [113]. 

Cellulose derivatives-based hydrogels were studied as transdermal drug-delivery systems due to their excellent properties, including: (i) their simple application, (ii) reduction of the systemic side effects, (iii) avoidance of the liver first-pass effect, and (iv) capacity to provide a improved feeling for the skin in comparison with other conventional unguents and patches [9]. 

Skin penetration is decreased when using high molecular weight or charged active substances, due to the structure and physicochemical properties of the drug. As a result, in the transdermal administration of drugs, attempts have been made to improve drug delivery by using various physical penetration techniques, such as iontophoresis, sonophoresis, electroporation and laser irradiation [8]. In the case of hydrogels based on cellulose derivatives, different studies have been reported related to the transdermal delivery of different drugs, by using iontophoresis alone or in combination with other physical or chemical techniques.

For instance, the clinical efficiency of iontophoresis has been proved for a non-steroidal anti-inflammatory drug (celecoxib) investigated on several gel formulations with different gelling agents, such as sodium alginate, CMC, HPMC, and carbopol 934P [114]. Hydrogel obtained from hydroxypropyl methylcellulose (HPMC) has been shown to have a higher spread and also an improved skin retention capacity, along with a release of the highest percentage of celecoxib (41.5%) after 5 h. Following these observations, it was considered that this formulation is optimal for iontophoretic studies. In addition, ex vivo studies have confirmed that the iontophoretic transport of the drug from hydrogels to the rat skin was twice as high as the passive flow [114]. 

A study of the transdermal release of metoprolol tartrate was performed, where it was shown that the combination of iontophoresis with sodium lauryl sulfate (SLS) allowed the release of a larger amount of the drug and also allowed its retention in the skin [115]. The gel formulations used were prepared from methyl cellulose (5% *w/v*), HPMC (2% *w/v*) and carbopol (1.5% *w/v*), and while SLS was used as permeation enhancers, dimethyl formamide (DMF), n-methyl-2-pyrrolidone (NMP) and polyethylene glycol 400 (PEG). It was shown that the permeability of metoprolol was not influenced by the type of the polymer selected for the making of the gel, through the obtained flow values of 4.59–5.89 µg/cm^2^/h (passive processes) and 37.99–41, respectively, 57 µg/cm^2^/h (iontophoresis). An increase in transdermal flow was also observed (approximately 3–5 times) through the use of chemical enhancers (SLS, DMF, NMP and PEG 400 at 5% *w/w*). In conclusion, this study suggests that the duration of treatment in topical/transdermal drug administration could be extended for existing and new transdermal drugs [115]. 

**Figure 11:**
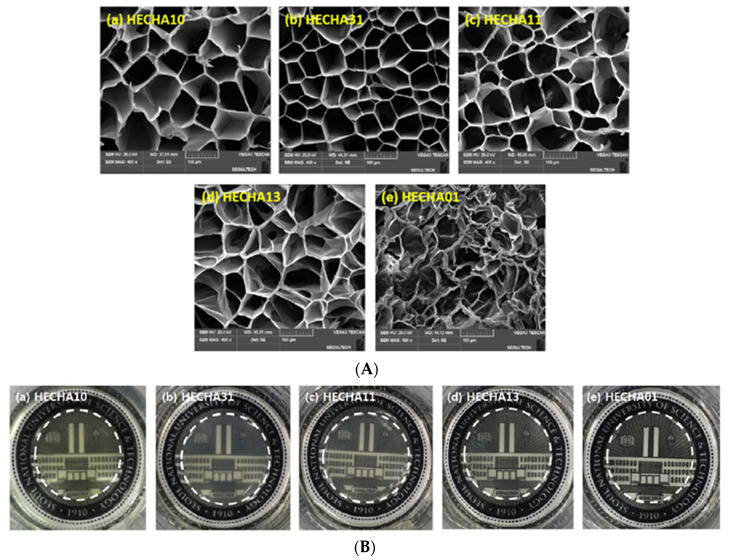
(**A**) Scanning electron microscope (SEM) images of cross-sections of the hydroxyethyl cellulose/hyaluronic acid (HECHA) hydrogels (the initial magnification was 400); (**B**) images of HECHA hydrogels: the white dotted line indicates the shape of the hydrogel; all hydrogels appeared transparent, and an increase of HA ratio was associated with greater transparency [116].

A pH-responsive hydrogel made from HEC and HA was prepared by the Michael addition reaction, using divinyl sulfone as the crosslinking agent [116]. HA has skin compatibility and pH functional groups and HEC serves as scaffold to build hydrogels with varied HEC:HA mass ratio (Figure 11).

The hydrogel thus prepared has been used in the treatment of acne, as a transdermal system for the administration of the drug (isoliquiritigenin), when it has been shown to have a high permeability of the skin. The hydrogel HECHA13, i.e., HEC:HA mass ratio of 1:3, was found to have optimal rheological and adhesive properties, and the drug-release efficiency was greater than 70% at pH 7 [78,116,117].

Among the cellulosic derivatives, the CMC shows better bioadhesive properties and a strong adherence to biological surfaces, in comparison with most of the nonionic cellulose derivatives. These properties make CMC an attractive platform for transdermal and transmucosal applications [72]. 

Another study evaluated the hydrogels based on CMC or MC in the transdermal administration of Atenolol (a beta-blocker used in high blood pressure and heart-related chest pain) through the abdominal skin of a rat. Iontophoresis was studied alone or using various chemical enhancers, such as L-menthol and Tween 20, when it was observed that compared to passive release, it increased the transport of Atenolol through rat skin. Thus, it has been shown that hydrogels based on cellulose derivatives provide a sustained release of Atenolol and are more suitable than the solutions. Thus, for hydrogels based on CMC, the Atenolol flux by using iontophoresis combined with L-menthol was increased to 25.8 g/(min·cm^2^) compared with 18.033 μg/(min·cm^2^) in passive diffusion; and for MC gels, the Atenolol flux was increased to 25.43 μg/(min·cm^2^) compared with 17.766 μg/(min·cm^2^) in passive diffusion) [118].

Ionic crosslinking of CMC with polyethyleneimine (PEI) has proven to be the appropriate solution for the preparation of syringeable formulations, which at body temperature form time-stable gels over long periods (two weeks). Hydrogel CMC-PEI systems have been studied in vitro and in vivo. In this regard, a sustained release of fluorescein isothiocyanate-labeled bovine serum albumin (BSA-FTIC) for 9 days was observed, and in the case of subcutaneous administration to rats, maintenance of plasma proteins for 16 days, was recorded [119]. 

Although numerous studies have been performed on transdermal drug delivery systems, there is still an important issue that needs to be considered, namely improving skin permeability and thus increasing the bioavailability of drugs. Studies to date have focused on breaking the barrier function of the skin, with little emphasis on the resistance of the carrier network for drug diffusion.

In this regard, a new hydrogel drug carrier for the transdermal purpose was developed by adding CMC to Poloxamer 407 (P407). This hydrogel was chosen to study the diffusion behavior of gallic acid (GA, model drug) by analyzing the concentration of the drug that penetrated the skin of the pig’s ear, selected as a permeation membrane. It was found that the presence of CMC clearly improves the porous structure of hydrogel matrixes: 4% CMC slightly decreases the pore size from 4.813 µm to 4.343 µm and causes a remarkable increase in the pore number fraction by around 10-fold compared to a matrix without CMC. A significant increase in drug permeability across the skin was also demonstrated, which is validated by the higher values of apparent permeability coefficient (P_app_) and supported by the porous structure of P407 hydrogel matrix after the addition of CMC [120].

Another study focused on the preparation of a thermo-responsive hydrogel based on CMC and gelatin, which was used in transdermal drug delivery, as a lidocaine delivery system. The hydrogel microspheres have a mean particle size diameter ranged from 5.89 to 14.60 μm depending on the gelatin content. All hydrogels outline rapid release of lidocaine during the first hour with steady state conditions observed in the next 3 h. The CMC/gelatin 1:1.6 hydrogel released the highest amount of Lidocaine (32.3% in 1 h), having the smaller particle sizes with a greater surface area [78].

### 4.4. Cellulose-Based Hydrogels for Ocular Administration

A major issue in ocular therapy is maintaining an adequate concentration of the drug for a certain period of time, in order to obtain the appropriate pharmacological response [121]. For this, an attempt was made to use viscosity-enhancing agents (cellulose derivatives), mucoadhesive polymers (polysaccharides) and in situ hydrogel production systems, so that ocular drugs are administered in an optimal concentration [7,122,123]. 

As cellulose derivatives used in topical ophthalmic preparations can be listed: methylcellulose (MC), hydroxyethyl cellulose (HEC), hydroxypropyl methylcellulose (HPMC), sodium carboxymethylcellulose (NaCMC), which have remained the most widely used polymers to date [109,124].

In order to make an ophthalmic solution that can form a gel in situ, a gelling agent (alginate) and a viscosity-enhancing agent (HPMC) have been used. Gatifloxacin, a broad-spectrum antibacterial agent, was used as a medicine to treat eye infections. The gel made of alginate/HPMC, formed behind the scenes, when instilled as a solution in the form of drops, allowed the release of the drug for 8 h, with a much better effectiveness than in the case of gels formed either from alginate or HPMC. The same behavior was also observed in the study of pre-corneal retention time, when the formulation prepared from alginate and HPMC had a longer retention time than the other two formulations. Thus, a 2.6-fold improvement was achieved by adding alginate/HPMC to the control; also, the elimination of the formulations from the pre-corneal area was delayed, the t_1/2_ values being 4.0- to 19.9-fold greater than the ophthalmic solution. Furthermore, the remaining activity of alginate/HPMC formula after 10 min was 78.66%, which was almost 6.3-fold that of the eye drops. All these results demonstrated that alginate/HPMC formula can be considered a viable alternative to conventional eyes drops [11].

A new in situ gelling system based on sodium alginate and MC was realized, with possible applications as sustained ophthalmic drug-delivery system [125]. It was shown that at a pH of 4.7, the system was in sol form, while to a pH up to 7.4, this suffered a rapid sol-gel transition. The drug used in this study was sparfloxacin and the evaluation of the formulation proved that can be used as a sustained drug release system. In addition, the formulation was tested with good results for corneal permeation on a goat eye.

Thermo-responsive hydrogels were obtained by embedding α-cyclodextrin (α-CD) in a hydrophobically changed gel based on hydroxypropyl methylcellulose (HM-HPMC). The prepared HM-HPMC/α-CD gel showed a reversible sol-gel transition at the physiological temperature, while the original HM-HPMC (without α-CD) showed temperature dependence. Moreover, HM-HPMC/α-CD gel also showed a quick gelation on the ocular surface and a significant improvement related to the absorption of the ocular drug (diclofenac sodium) [124].

### 4.5. Cellulose-Based Hydrogels in Nasal Delivery

Carriers of active ingredients aimed at the nasal release of drugs should implicitly to facilitate the increase of the nasal residence time of these ingredients. 

Hydrogels, through their excellent properties, such as increasing the contact time between the mucosa and the drug, increasing the concentration of the drug at the site of deposition and facilitating the permeability of the drug through the mucosa by opening tight junctions between epithelial cells, are recommended for their use as release systems nasal medication [13,126]. As for the mucoadhesive polymers that can be used in the preparation of hydrogels, they must meet certain minimum conditions in order to be used in nasal release formulations, conditions that allow daily use and sustained release of drugs, such as: (i) to does not irritate the mucous membrane, (ii) adheres rapidly to tissue and (iii) has a site-specificity [14]. Mucoadhesion can be accomplished by using different bioadhesive excipients, as microcrystalline cellulose, HPC, HPMC, chitosan, starch, and carbopol.

As for cellulose-based hydrogels with applications in intranasal administration of the drug, they are made from different cellulose derivatives, such as CMC, HPMC, MC or EC. These cellulose derivatives have properties designed (i) to facilitate the sustained release of the drug, by their high viscosity after their swelling in the nasal cavity, (ii) good mucoadhesive properties, which improve the intranasal absorption of drugs and (iii) increased bioavailability [3]. As proof of the existence of these special properties imprinted by the presence of cellulose derivatives, there are several references that indicate that cellulose is effective in increasing the intranasal bioavailability of all types of drugs, both hydrophobic and hydrophilic [14].

Among cellulose derivatives, CMC is indicated as a system of administration of drugs conducive to mucosal tissue, following the study of nasal release of apomorphine (used to regulate motor responses in Parkinson’s disease) [65]. The CMC powder formulation allowed the drug to be incorporated and showed a sustained nasal release, much better than when using a starch-based formulation. The percentages of the formulations cleared from the nasal cavity, 30 min after insufflations, were 47% for lactose, 26% for lactose/apomorphine, and 3% for CMC/apomorphine; after 3 h, the percentages were 70% for lactose, 58% for lactose/apomorphine, and 27% for CMC/apomorphine, respectively.

### 4.6. Cellulose-Based Hydrogels in Vaginal Delivery

Intravaginal drugs are used to treat sexually transmitted diseases, the human immunodeficiency virus and various vaginal infections. Hydrogels, with their advantage of controlled and sustained drug release, are of real interest and have proven to be particularly effective in this area.

**Figure 12:**
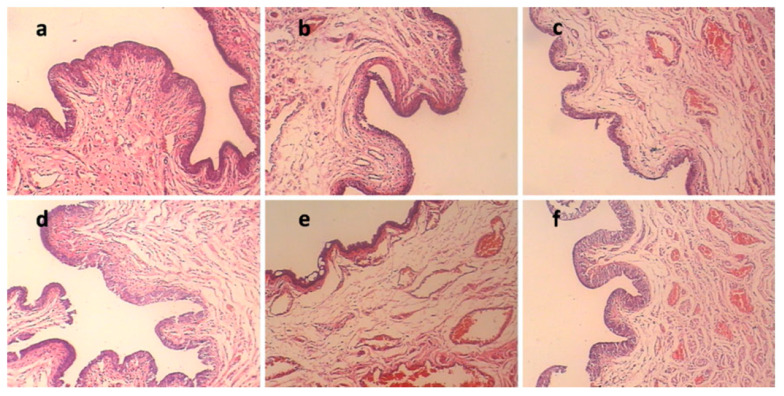
Pathological photos of rabbits’ cervicovaginal tissues after intravaginal application of 0.9% NaCl (**a**,**d**), hydroxyethyl cellulose (HEC) gel (**b**,**e**) and methylcellulose (MCS) hydrogel (**c**,**f**) for 1 day (**a**–**c**) and 7 consecutive days (**d**–**f**), respectively [23].

An example of this is the thermosensitive hydrogel prepared from stearic acid modified methylcellulose (MCS), which at a temperature close to that of human body showed sol-gel transition performance (Figure 12) [23]. Also, the hydrogel had a good biocompatibility, similar to that of the hydrogel obtained from HEC, proved from the cytotoxicity studies (the cell viability of MCS and HEC were still 83% and 80%, after 24 h). 

In order to establish the controlled release capacity of the drugs, the release of tenofovir, a drug used to treat human immunodeficiency virus (HIV), was investigated. Hydrogel has been shown to be a successful system for the intravaginal release of antiviral drugs, due to a 10 h release of tenofovir, a period of time much greater than the cases of controlled release of HEC or poloxamer 407-based hydrogels (e.g., for HEC gel was observed a burst release of tenofovir, about 53% of the loaded-drug were released within the first 0.5 h).

### 4.7. Cellulose Based-Hydrogels as Delivery Systems in Cancer Therapy

The classic methods of treating cancer are oriented towards several therapies, namely: surgical operations, chemotherapy and irradiation [60]. Chemotherapy, the most widely used therapy, involves the administration of drugs with high toxicity, but unfortunately with low specificity, too, which kill not only cancer cells but also normal ones. Thus, systemic administration of chemotherapeutic drugs face the following limitations: (i) reduced selectivity to tumor cells, and unwanted damage to healthy organs due to uneven distribution of the drug throughout the body (ii) the need for repeated administration due to ineffective dosing of the drug at the tumor site [127], (iii) poor aqueous solubility and reduced bioavailability of chemotherapeutic agents due to their hydrophobic nature and last but not least (iv) chemotherapeutic resistance that limits the efficacy of anticancer drugs [60].

Hydrogels are in many respects, suitable candidates as carriers in cancer therapy for delivering anti-cancer drugs in a controlled and targeted manner [127]. Hydrogels in various forms including injectable formulations [128,129], nanoparticles [130] and nanocomposites [131] have been explored for controlled and sustained delivery of chemotherapeutic agents. 

**Figure 13:**
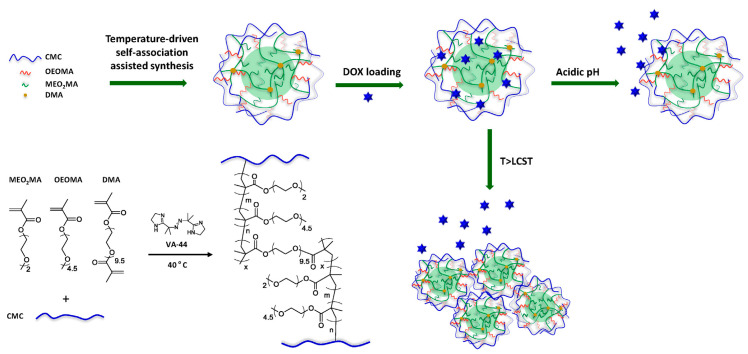
Synthesis of dual temperature/acidic pH-responsive dual temperature/acidic pH-responsive nanogels (DuR-BNGs) via temperature-driven self-association method and their dual stimuli-responsive doxorubicin (DOX) release [132].

In particular, stimuli-responsive hydrogels are extensively investigated in this respect, the chemotherapeutics being easily entrapped in the hydrogel matrix followed by their release under an external stimulus [25]. The use of thermo-responsive hydrogels as administration systems for chemotherapeutic agents started from the fact that they can be injected directly into the tumor, in liquid form, and then, in the presence of body temperature, become gel [127]. pH-responsive hydrogels have also been researched for anticancer drug delivery benefiting from the pH difference between the tumor environment (pH = 5–6) and physiological pH of 7.4 (Figure 13) [132]. 

Another important class of polymeric systems used to treat cancer are the magnetic hydrogel nanocomposites, which incorporate magnetic nanoparticles, such as iron oxide (Fe_3_O_4_) or maghemite (γ-Fe_2_O_3_). This is a new class of stimulus-receptive hydrogel, which has the ability to be controlled remotely by a magnetic field and are used for the controlled release of drugs directly to cancer cells [133]. 

Nanogels, hydrogels with dimensions in the submicron range, are receiving considerable attention as controlled drug-delivery systems for cancer therapy, due to the special properties obtained by their size, such as: (i) improved bioavailability, (ii) biocompatibility, (iii) a large surface to multivalent bioconjugation, (iv) targeted drug release, (v) lighter intracellular permeability, (vi) high stability and (vii) adjustable particle size [134,135,136]. Of particular interest are nanogels prepared from polymers receptive to external stimuli, as potential materials for the development of more effective treatments for disease. These are intelligent drug-delivery systems, which not only detect but also react directly to pathophysiological conditions [135]. The structural stability of nanogels and the biological characteristics of the tumor environment were the arguments behind the development of studies of cellulose-based nanogels for the administration of anticancer drugs [60].

Well-defined dual temperature/acidic pH-responsive nanogels (DuR-BNGs) were synthesized by crosslinking polymerization via the temperature-driven self-association method in aqueous solution (Figure 13) [132]. 

CMC contains pendant COOH groups that promote the release of encapsulated anticancer drugs in acidic tumor tissues, namely pH of 5.0–6.5 compared with pH 6.5 in extracellular environments. On the other hand, grafted copolymers based on oligo(ethylene oxide) are heat-sensitive polymers with a strong response (drug release) in the field of temperatures close to body temperature. Moreover, the release of encapsulated drugs from DuR-BNG also occurs when both stimuli act, a higher temperature and an acidic pH. In vitro studies demonstrate that DuR-BNG is a system that can be used successfully in releasing drugs receptive to dual stimuli used in cancer therapy [132].

Cellulose-based hydrogels containing functional inorganic nanoparticles have improved properties, such as conductivity, photoluminescence, and better mechanical properties, catalytic and magnetic properties, which make them suitable for various biomedical applications [137]. 

**Figure 14:**
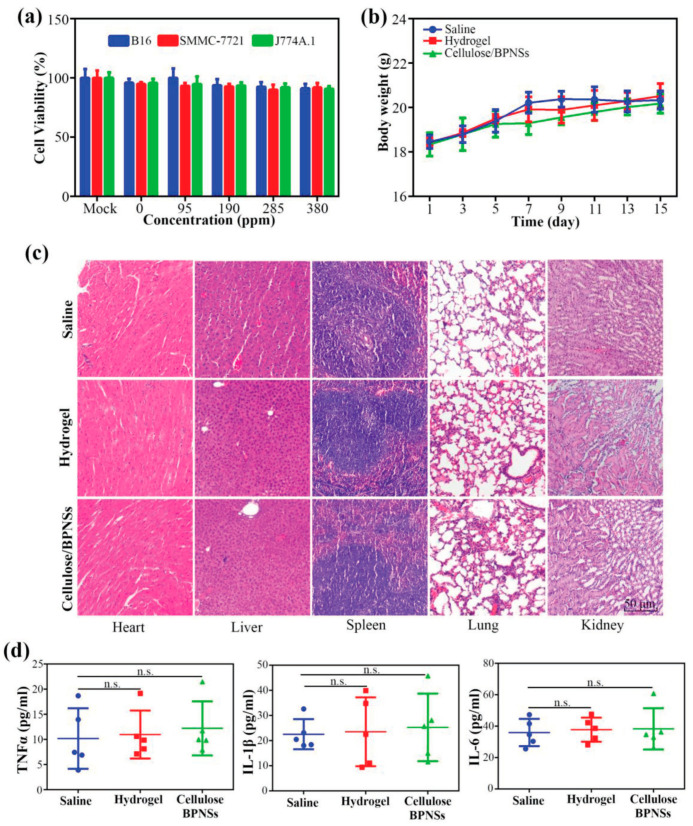
Toxicity assays: (**a**) In vitro toxicity assays. Relative cell viability of B16, SMMC-7721, and J774A.1 cells after incubation with Mock (no treatment) and hydrogels without black phosphorus nanosheets (BPNSs) or with BPNS concentrations of 95, 190, 285, and 380 ppm for 24 h; (**b**,**c**) in vivo toxicity assays after subcutaneous injection of 100 μL of saline, hydrogel, or cellulose/BPNSs; (**b**) body weight was monitored on days 1, 3, 5, 7, 9, 11, 13, and 15; (**c**) hematoxylin and eosin (H&E) staining of the heart, liver, spleen, lung, and kidney on day 15; (**d**) Immunotoxicity assays in vivo for protein levels of TNFα, IL-1β, and IL-6 in the serum of C57BL/6 mice after indicated treatments [138].

Nanocomposite hydrogels obtained from cellulose and black phosphorus nanosheets (BPNS), following a light green chemical crosslinking reaction, showed special properties due either to the presence of BP (excellent photothermal properties) or due to the presence of cellulose (good mechanical properties). In addition, they have proven biocompatibility for cells and organs, which has qualified them as injectable photothermal systems, effective in treating cancer in mice (Figure 14) [138].

Another hydrogel nanocomposite that can be used in the treatment of cancer was made from a cellulose derivative (CMC), from which the hydrogel was obtained, where nanoparticles were incorporated in the form of quantum graphene dots (GQDs). GQDs have been shown to have a much lower cytotoxicity than other inorganic quantum dots, which by degradation can release toxic metal ions. The nanocomposite hydrogels thus obtained showed an improvement in the degree of swelling and in the degradation process, presented reduced toxicity to blood cancer cells (K562) and the possibility to administer of pH-sensitive drugs. The incorporation and release of a model drug, doxorubicin (DOX), used to treat cancer, was also studied. The incorporation of GQD in CMC films induced a pH sensitivity of the system and consequently caused the prolongation of DOX release. The positive results of cytotoxicity tests, developed on blood cancer cells (K562), demonstrated the abilities of the prepared nanocomposite DOX/CMC/GQD as a long-lasting and highly effective anticancer agent [139]. 

**Figure 15:**
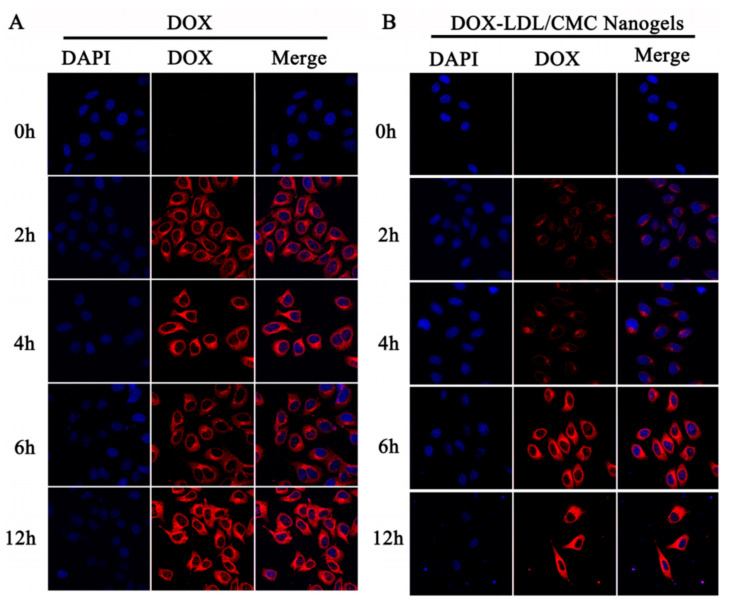
Confocal laser scanning microscopy (CLSM) images showing intracellular uptake of doxorubicin (DOX) (**A**) and DOX-loaded low-density lipoprotein (LDL)/carboxymethylcellulose (CMC) nanogels (**B**) by human hepatocellular liver carcinoma cell lines (HepG2 cells). DOX dosage was 4.0 g/mL. It was investigated qualitatively by CLSM [140].

Well-defined low-density lipoproteins (LDL)/CMC nanogels were successfully fabricated as spherical nanoparticles and used to encapsulate the DOX. The nanogels had a polysaccharide surface that made the nanogels stable in various pH conditions (3.0–10.0). In vitro studies showed that drug release was slow at the pH characteristic to physiological conditions (7.4), while at a pH of 6.2 drug release was achieved in larger quantities. Also, the use of confocal laser scanning microscopy (CLSM) and flow cytometry measurements showed that DOX-embedded LDL/CMC nanogels decreased endocytosis in human hepatocellular liver carcinoma cell lines (HepG2 cells), a fact which recommended them as drug-delivery systems in cancer therapy (Figure 15) [140].

Redox-responsive nanogels, prepared by methacrylation of carboxymethyl cellulose, were used as drug carriers, for doxorubicin (DOX). Non-invasive live body fluorescence imaging technology showed that nanogels could passively target to the tumor area by the enhanced permeability and retention (EPR) effect. An in vivo study in liver mice carrying H22 tumors showed that the nanogels, in which DOX was incorporated, have a long circulation time and that the drug released recorded a strong concentration at the site where the tumor was located, thus demonstrating a high antitumor efficacy compared to the free drug [141].

Kim and coworkers [142] conceived a thermo-responsive system, for high-efficiency delivery of docetaxel (DTX), based on a low molecular weight methylcellulose (LMw MC) and Pluronic F127. The use of a non-ionic copolymer surfactant (Pluronic F127) has been shown to be effective in increasing the solubility of lipophilic drugs such as DTX. DTX is encapsulated in the hydrophobic core of the surfactant, resulting from the presence of hydrophobic poly (propylene oxide) in the chain. The combined gel (MC)/micelle (Pluronic F127) system for local DTX delivery proved to be very effective compared to the free DTX formulation obtaining complete tumor remission at a high dose of DTX administration without side effects by sustained release of DTX directly into the tumor region.

Table 3 summarizes data on the use of cellulose-based hydrogels as drug delivery systems in cancer therapy, highlighting the influence of their physical properties on drug delivery efficiency.

### 4.8. Injectable Cellulose-Based Hydrogels in Drug Delivery

Injectable hydrogel formulations are particularly appealing and currently under investigation in the biomedical field, being proposed as a platform for the delivery of therapeutics [36]. Injectable hydrogels are superior to pre-formed hydrogels, primarily due to their ability to fill and cover spaces of any shape and secondly because they do not require a surgical procedure for implantation [143]. They can be delivered to the body either through a catheter or directly by injecting the affected area [144]. Stimulus-sensitive injectable hydrogels are known to have a high affinity for body fluids and are capable of changing sol–gel transitions in response to various external stimuli, such as temperature, pH, enzyme, light or magnetic field [145].

In order to be applied in clinical trials, injectable hydrogels must have several essential properties, such as biocompatibility, biodegradability and non-toxicity, must be stable, and must have superior mechanical and viscoelastic properties, in order to be able to withstand possible deformations that appear in the body. Regarding the viscosity, it is important that the precursor aqueous solutions have adequate shear-thinning properties that allow them to be easily injected [39]. An essential factor of injectable hydrogels is their biodegradability so that, after degradation, the products obtained are eliminated from the body, without surgery to remove them [146].

Polysaccharide-based injectable hydrogels are extremely advantageous and have a variety of biomedical applications, in drug delivery and tissue engineering [143]. Due to their special properties, such as their biocompatibility and biodegradability, cellulose derivatives are successfully used as injectable materials for biomedical applications. A new hydrogel based on oxidized carboxymethylcellulose (OCMC) and *N*-succinyl-chitosan (NSC), gelled in situ at a physiological pH and temperature, without the addition of crosslinking agents, has been shown to be non-toxic, to have a good cellular viability (more than 93% cells viability after 48 h) and can be used in the administration of drugs [146]. In this sense, a sustained release of bovine serum albumin (BSA) was recorded both through the diffusion-controlled mechanism and through the degradation-controlled mechanism (18% to 44% BSA released in the first 10 h, depending on OCMC/NSC ratios). In addition, it was observed that BSA release was not influenced by the degree of cellulose oxidation.

Injectable hydrogels are used also in cancer therapy, in order to ensure a controlled and targeted release of the drug, without exceeding the toxicity limit, which classic chemotherapy treatments often exceed [39]. For effective regression of primary tumors or for the eradication of tumor cells remaining after surgery, the local injection of chemotherapeutic drugs is a much more beneficial solution than systemic administration, by a large local accumulation of drugs in the tumor region, and by reducing side effects [142].

## 5. Conclusions

Hydrogels are of particular interest in the drug delivery applications, due to the possibility to choose the proper type of polymer, to design matrices with spatial and temporal control over the release of various therapeutic agents, and to expand the range of drugs used, which gives them therapeutically essential outcomes.

The properties of cellulose, like abundancy, low cost, biocompatibility, biodegradability, cytocompatibility, various ways of derivatization which can be customized according to needs, make cellulose-based hydrogels particularly attractive for medical applications. Moreover, these can be easily designed (by selecting the suitable polymer) to imprint the ability to respond to different external stimuli (physical, chemical, or biological stimuli) with properties to protect, target, and locally deliver drugs in a controllable manner. However, there are some issues which should be overcome, related to the administration route of active pharmaceuticals, such as: (i) reduced mechanical strength, (ii) a very fast (burst) drug release, (iii) the fact that hydrophobic drugs have a limited delivery, (iv) a slow response of stimuli-sensitive hydrogels, (v) the possibility of drug reaction, etc. Another dilemma is related to hydrogel degradation, which is used for the controlled release of proteins, growth factors, and genes, depending on the site for which is intended to be used. Thus, there is the possibility of using hydrogel formulations for the administration of drugs orally or transdermally, at which time it is not necessary to degrade the hydrogel. However, if the formulations are used in different areas of the body (in situ), then they need to be biodegradable, in order to be eliminated without surgical operations.

Taking into account all these observations it is easy to observe the capacity of cellulose-based hydrogels, that (i) can respond to the environmental modifications, (ii) can provide an adequate release profile (continuous or pulsatile), (iii) can be adhesive to a dynamic surface, in the treatment of skin wounds, (iv) can resist at compression and scratching, (v) can be degraded after the drug is exhausted, in order to avoid surgical removal, and (vi) can have desirable therapeutic outcomes, as required.

As presented in the review, there are already several cellulose-based hydrogels for drug-delivery applications, with clinical uses, but there are always possibilities to improve their properties and to enlarge their application areas. Significant progress has been made in improving the properties of customized hydrogels by introduction into the hydrogel structure of response mechanisms, which can only be triggered by the target physiological environment. The current disadvantages and limitations of cellulose-based hydrogels will be overcome through continuous research focused on the development and improvement of these, in order to obtain drug-delivery systems with promising results for the treatment of several diseases.

## Figures and Tables

**Table 1:** pH profile along the gastrointestinal tract.

Location		pH		
		Average pH [16]	Children [17]	Adults [17]
Oral cavity		5.8–7.4	-	-
Esophagus		5.0–6.0	-	-
Stomach	Fasted conditions	1.5–2.0	1.5	1–2.5
	Fed conditions	3.0–5.0	-	-
Small intestine	Duodenum	-	6.3–6.4	5–6.5
	Jejunum	5.0–6.5	6.6–7	5.8–7.7
	Ileum	6.0–7.5	7.3	6.6–7.7
Large intestine	Cecum	-	5.8–5.9	6–6.5
	Ascending colon (right colon)	6.4	5.9–6.5	4.6–6.8
	Transverse colon (mid colon)	6.0–7.6	5.3	4.6–7.1
	Descending colon (left colon)	6.0–7.6	6	5.5–7.4
	Rectum	-	6.5	5.3–7.4

**Table 2:** Cellulose derivative-based hydrogel as drug-delivery systems.

Cellulose Derivatives	Drug	Treatment	Ref.
Methylcellulose (MC)	Cyclosporine A	Sustained brain delivery	[84]
	Erythropoietin	Delivery to the brain in order to endogenous stem cell stimulation after stroke	[85]
Carboxymethylcellulose (CMC)	Amoxicillin	Excellent antibacterial agent against gram-positive *Staphylococcus aureus*	[86]
	Acyclovir	Controlled drug delivery systems	[87]
	Diclofenac	Skin wounds	[1]
	Nonivamide	Improved skin permeation and distribution	[88]
	Berberine	Protect postsurgical tissue and perform a controlled drug release	[89]
	Propolis	Wound healing	[90]
Hydroxyethyl cellulose (HEC)	Eugenol	Efficient bacteriostasis against *Escherichia coli*	[91]
	Isoliquiritigenin	Transdermal delivery system	[92]
Hydroxypropyl cellulose (HPC)	Lidocaine	Promote a systematical and controlled drug	[93]
Hydroxypropyl methylcellulose (HPMC)	Etoricoxib	Chronic or acute illness	[94]
	Fluconazole	Skin fungal infections	[95]
	Mepivacaine	Relieve local pain and perform a controlled drug release	[96]
	Propranolol	Improve percutaneous penetration	[97]

**Table 3:** Physical properties of cellulose-based hydrogels and their drug-release efficiency as delivery systems in cancer therapy.

Cellulose-Based Hydrogels	Physical Properties	Drug Release Efficiency	Ref.
CMC dual temperature/pH response nanogels (DuR-BNGs)	− LCST = 32 °C at 4.6 mg/mL; − Diameter = 10.5 nm; − Colloidal stability in pH ranges 5.5–7.0 (at room temperature) and in the presence of proteins; − Non-toxicity at C > 1.0 mg/mL (>90% of cell viability).	− Rapid DOX release at pH 5.5 (cancer cell environment), compared with pH 7 (physiological condition).	[132]
Cellulose/BPNSs nanocomposite hydrogels	− 3D networks with excellent flexibility and elasticity; − Good photo-thermal and thermodynamic stability; − Mechanical properties: − Elastic modulus = 30 ± 2.1 Pa; − Strength at break = 13.9 ± 1.6 kPa; − Strain at break = 50.0 ± 4.9%; − No detectable toxicity; − High biocompatibility and biosafety.	− Cellulose hydrogels (without BPNSs), but with near-infrared (NIR) irradiation showed a slightly decreased tumor volume; − BPNSs addition had no effect on tumor growth without NIR irradiation, even at 380 ppm BPNS; − The tumor exhibited significant regression (was almost totally killed) with NIR irradiation.	[138]
CMC/GQDs nanocomposite hydrogels	− GQDs nanoparticles with uniform circular shape; − Narrow range distribution of particle size from 4 to 6 nm; − Photoluminescent properties; − Mechanical properties: 20% GQDs slightly increased the tensile strength; − Suitable flexibility.	− Fastest rate of DOX release − in 10 h (pH 4.5 and 7.4); − DOX release decreases with GQDs concentration: − CMC/GQDs 30% ~ 35%; − CMC/GQDs 20% ~ 25%; − CMC/GQDs 10% ~ 10%.	[139]
LDL/CMC nanogels	− Spherical shape; − Average diameter of 90 nm; − Negative zeta potential: −35 mV; − Polysaccharide surface stable in various pH conditions (3.0–10.0); − High DOX encapsulation efficiency (∼98%).	− Sustained DOX release profile at pH 7.4 and 6.2; − Initial burst for 1 h, followed by sustained release up to 14 h; − Cumulative releases of DOX at pH 6.2 and 7.4 were 62.29% and 46.04%, respectively.	[140]
Methacrylated/ CMC nanogels	− Spherical morphology; − Diameter of about 192 nm; − Negative surface potential: −25.7 ± 1.6 mV; − Stable against high salt concentration, but not in environments which contain glutathione (GSH); − Nontoxic and cytocompatible; − High DOX encapsulation efficiency (83%).	− Without GSH (pH 7.4), only about 20% of the DOX loaded is released within 24 h, and no tendency of further release is observed thereafter until 96 h; − DOX release amounts at pH6.5 and 4.5: 45% and 70%, respectively, within 96 h; − With 10 mM GSH in PBS (pH = 7.4), 70% of the loaded DOX is released in the initial 24 h and this value becomes 80% in 96 h.	[141]
LMw MC/ Pluronic F127 hydrogels	− 3% AS provided optimal gelation time at 37 °C and appropriate viscosity at room temperature; − Viscosity fluctuations between 25–37 °C (with the return to the initial value at a temp. of 25 °C, without losing physicochemical properties).	− Sustained cumulative release of DTX for over 38 days: − less than 20% release (24 H); − approx. 50% (7 days), − a very slow release up to 95% (38 days).	[142]

DOX—doxorubicin; LCST—low critical solution temperature; BPNSs—black phosphorus nanosheets; GQDs—quantum graphene dots; LDL—low-density lipoproteins; GSH—glutathione; LMw MC—low-molecular-weight methylcellulose; AS—ammonium sulfate; DTX—docetaxel.
